# Attentional deficits in fibromyalgia: an ERP study with the oddball dual task and emotional stroop task

**DOI:** 10.1186/s40359-024-01601-3

**Published:** 2024-02-29

**Authors:** Susana Cardoso, Carina Fernandes, Fernando Barbosa

**Affiliations:** 1 Research Center in Sports Sciences, Health Sciences and Human Development (CIDESD), University of Maia, Avenida Carlos de Oliveira Campos-Castêlo da Maia, 4475-690 Maia, Portugal; 2 Laboratory of Neuropsychophysiology, Faculty of Psychology and Education Sciences, University of Porto, Porto, Portugal; 3 Faculty of Human and Social Sciences, University Fernando Pessoa, Porto, Portugal; 4 Molecular Oncology and Viral Pathology Group, Research Center of IPO Porto (CI-IPOP) & RISE@CI-IPOP (Health Research Network), Portuguese Oncology Institute of Porto (IPO Porto)/Porto Comprehensive Cancer Center (Porto.CCC), Porto, Portugal

**Keywords:** Attention bias, Attentional deficits, Chronic pain, Oddball dual task, Emotional stroop task, EEG, Fibromyalgia

## Abstract

The present study investigated the neural correlates of attentional deficits in fibromyalgia through an Oddball Dual Task and an Emotional Stroop Task, both performed during EEG recordings. Thirty female participants were recruited, being divided into two groups: a group of patients with fibromyalgia (FM, *n* = 15, *M*_age_ = 51.87, SD_age_ = 7.12) and a healthy control group (HC, *n* = 15, *M*_age_ = 46.13, SD_age_ = 8.41). In the Emotional Stroop Task, the behavioural results showed that patients with FM had less hits and longer times reactions than healthy controls. These results were consistent with those obtained with our Event-related Potential (ERP) methodology, which evidenced that patients with FM had higher frontal latencies in the P200 time-window compared to healthy controls. Regarding the Oddball Dual Task, we found that patients with FM had lower P300 amplitudes than healthy participants. Moreover, we found that rare stimuli elicited higher P300 amplitudes than frequent stimuli for healthy controls, but this comparison was non-significant for patients with FM. Taken together, our results suggest that fibromyalgia may be associated to a reduced processing speed, along to reduced neural resources to process stimuli, mainly in distinguishing relevant (rare) and irrelevant (frequent) stimuli according to the goals of the task. Altogether, our results seem to support the hypothesis of generalized attentional deficits in FM.

**Supplementary Information:**

The online version contains supplementary material available at 10.1186/s40359-024-01601-3.

## Introduction

Fibromyalgia (FM) is a chronic pain condition with a variety of cognitive and affective symptoms [1]. It manifests with persistent widespread pain, fatigue, sleep disturbance, morning stiffness, depression, anxiety, and impaired cognitive functioning, mainly in the attention, memory, and executive functions [1–4]. It is estimated that FM affects between 2% and 4% of the general population, being nine times more common in women than in men [5].

There is extensive literature supporting the existence of cognitive impairment in fibromyalgia patients, which are more pronounced in highly demanding tasks, composed of stimulus-competitive activities. This type of dysfunction affects their performance in activities that require concentration, attentional control, and information management to adequately execute tasks in their daily lives [6–9]. Some studies examining sensory processing showed that patients with FM have lower thresholds for painful stimuli, as well as earlier detection of other somatosensory stimuli [10]. These indicators may be suggestive of a hypersensitivity to pain and to the detection of other somatosensory stimuli in patients with FM that may be modulated or maintained by the characteristic presence of a phenomenon of hypervigilance in pain perception in these patients [11, 12].

Hypervigilance to pain has been defined as an enhanced and selective attentional response among patients with chronic pain, resulting from both automatic and controlled processes that arises when pain is appraised as a threatening stimulus. This increased attentional response activates the fear system, which subsequently triggers catastrophic thoughts that direct the individual’s concern towards escape and avoid pain [13]. According to this theory, patients with chronic pain tend to appraise bodily sensations as dangerous or threatening.

Recent studies proposed the existence of a generalized hypervigilance in fibromyalgia, not only restricted to stimuli with painful content, but also to stimuli with neutral content [14]. Studies assessing neural activity have been supporting this hypothesis [15]. Moreover, two studies showed an association between alterations in pain threshold and tolerance to generalized hypervigilance to nociceptive stimuli [16–18]. They demonstrated that, despite the absence of peripheral lesions of nerves, patients had altered pain thresholds to hot and cold sensations along with reduction in pain tolerance. Accordingly, the study conducted by [19] showed that people with fibromyalgia were more sensitive to pressure and to the presence of everyday auditory stimuli.

In line with this evidence, [15] analyzed Event-related Potentials (ERPs) time-locked to series of 1000 Hz auditory tones at different intensities (60, 70, 80, 90 and 105 dB) and found that patients with FM, compared to healthy controls, had lower latency and higher amplitude of the N1-P2 auditory component for the most intense stimuli (105 dB), suggesting that FM patients may have an alertness to stimuli of various perceptual modalities. Using stimuli of visual modality, [14] used an emotional Stroop paradigm to study the phenomenon of generalized hypervigilance in FM. The group of patients showed a slowing in the colour naming task, associated only with words in the neutral category.

Taking together, these results suggest that fibromyalgia is characterized by the presence of a generalized hypervigilance that is associated with increased sensory processing at the level of the peripheral [16–18] central nervous system [14, 15]. However, these the hypothesis of hypervigilance is far from being consistent, considering the results of other studies that found no evidence to support this theory [20–23]. For instance, [24] found interference effect towards words with negative valence in FM patients compared to a control group. As the severity of pain explained the higher variance of interference of negatively stimuli, this result suggests that patients with FM may have an attentional bias to negative information instead of a generalized attention to emotional and neutral stimuli from the environment.

In addition to the hypervigilance and attentional bias hypotheses, the results of other studies have pointed to the hypothesis of a generalized deficit of attention in patients with FM. For instance, when studying cognitive functions in patients with FM with an auditory oddball paradigm, [25] found an increased latency and reduced amplitude of the P300 component, interpreting them as an indicator of dysfunctions in cognitive abilities. A study [26] also found and reduced N2-P3 amplitudes in patients with FM compared to healthy controls, further showing that the P300 latencies correlated negatively with the total myalgia score of the patients. An investigation [27] found that the amplitude of the Mismatch Negativity obtained in the right hemisphere during an auditory oddball paradigm was lower in patients with FM than in healthy controls, being associated with a lower pain threshold.

Other studies seem to support the thesis of generalized attentional deficits in FM and augmented emotional processing of the target stimuli [28–30].

There are several studies that relate aspects of fibromyalgia to impaired cognitive functioning and other clinical variables. For example, a study [31] demonstrated that body mass index and pain severity explained the largest proportion of variance in performance on the executive functions of updating, change inhibition, decision making, and planning in people with FM. Another research demonstrated that cognitive impairment in FM is associated with alterations in cerebral blood flow responses during cognitive processing [32]. An interesting study concluded that the experience of pain during low-intensity somatosensory stimulation is more intimately related to attention, memory, and executive functions in FM than traditional measures of pain threshold and pain tolerance. Considering that the phenomena of hyperalgesia and allodynia -characteristic of FM- are pain responses to low-intensity stimulation, they suggest that the central nervous sensitization to pain hypothesis may be implicated in cognitive impairments in this clinical condition [33].

### The present study

In the present study, we aimed to explore how modulation of the emotional context might affect cognitive performance in patients with FM and matched healthy controls. To this purpose, our study included two experimental tasks– an Oddball dual-task and an Emotional Stroop Task - that were performed during EEG recordings. The oddball dual-task included tones with higher probability of occurrence and tones with lower probability of occurrence, which were presented simultaneously with emotional (pain-related) or neutral words. This task was designed to elicit two neural correlates of attentional processing, the N100 and P300. The N100 is a negative evoked potential appearing at around 100 ms after the onset of a stimulus and it is elicited by any discernible auditory stimulus. N100 has been related to the allocation of automatic attentional resources toward attended emotional stimuli [34]. That is, it has been associated with bottom-up attentional mechanisms. The P300 is a positive component that occurring after 300 ms at centro-parietal electrodes. Its amplitude is modulated by the probability of an event, by the personal relevance attributed to the stimuli, intentional engagement, and selective attention [35].

The Emotional Stroop Task was selected to investigate the effect of pain-related words as distractors, as the content of these words would capture attentional resources and delay the colour identification [36]. Thereby, the task consisted of the presentation of relevant (pain-related) and neutral words in different colours (blue, green, red, and yellow) on a black background. During this task we measured the P200 time-locked to the words, since this component appears to be modulated by the emotional significance of the stimulus [37–38]. Higher amplitudes and short latencies of P200 have been interpreted to indicate a negativity bias toward emotional information [39–40]. P200 is a salient positive wave over the vertex (Cz), with a peak latency of approximately 150 to 250 ms, elicited by visual auditory and somatosensory stimuli [41].

Through the inclusion of a pain-related and a neutral condition, these tasks allowed us to test the hypotheses of hypervigilance, attentional bias towards negative stimuli and generalized attention deficits. That is, if fibromyalgia is characterized by hypervigilance, we expect to find increased ERP components for FM patients compared to healthy controls in both pain-related and neutral condition. On the other side, if fibromyalgia is characterized by generalized attentional deficits, we expect to find reduced ERP components (P300) for FM patients compared to healthy controls in both pain-related and neutral condition. However, if fibromyalgia is characterized by attentional bias towards to negative information, we expect to find increased ERP components (P300) for the pain-related condition compared to neutral condition, while expecting similar amplitudes for both conditions in the control group.

## Method

### Participants

Thirty female participants were recruited, being divided in two groups: a group of patients with fibromyalgia (FM, *n* = 15) and a control group of healthy participants (HC, *n* = 15). FM patients were recruited from a National Association Against Fibromyalgia and Chronic Fatigue Syndrome (MYOS) and included a formal diagnosis of fibromyalgia based on criteria from ACR [1]–. Healthy participants were recruited from the community and included if they did not report history of chronic pain. Participants of both groups were included if they had Portuguese nationality, age between 25 and 65 years old, and more than four years of formal education. Participants of both groups were excluded if they had left hand as dominant, history of brain injury, neurological or psychiatric diagnosis, and uncorrected sensory or motor deficits. Both groups were statistically paired regarding education and age.

In the emotional Stroop task, data from two participants (one from FM group and other from HC group) were excluded from the ERP analysis due to excessive noise in the morphology of the ERPs. In the Oddball dual-task, data from one participant from the HC group was excluded from the ERP analysis due to excessive noise in the morphology of the ERPs.1 (Table 1).

**Table 1:** Characteristics clinics and socio-demographics of groups

	Fibromyalgia (*n* = 15)	Healthy controls (*n* = 15)	Statistic Test	Effect size
**Age (years)**				
Mean (SD)	51.9 (7.12)	46.1 (8.41)	*t* = 2.02	*d* = 0.74
Age range	38.0–64.0	33.0–58.0		
**Education % (n)**				
Primary	20.0 (3)	7.00 (1.00)	χ^2^ = 0.73	*Cramer’s V* = 0.73
Basic cycle	20.0 (3)	27.0 (4.00)		
High school	40.0 (6)	40.0 (6.00)		
Higher education	20.0 (3)	27.0 (4.00)		
Civil status % (n) *				
Married	93.0 (14)	53.0 (8.00)	χ^2^ = 0.03	*Cramer’s V* = 0.03
Single	0.00 (0)	33.0 (5.00)		
Widow	0.00 (0)	0.00 (0.00)		
Separated/divorced	6.70 (1)	13.0 (2.00)		
**Employment status % (n)**				
Active	47.0 (7)	80.0 (12.0)	χ^2^ = 0.23	*Cramer’s V* = 0.23
Never active	7.00 (1)	7.00 (1.00)		
Inactive for more than 1 year	40.0 (6)	13.0 (2.00)		
Inactive less than 1 year	7.00 (1)	0.00 (0.00)		
**Salary % (n)**				
More of 1.800 €	7.00 (1)	0.00 (0.00)	χ^2^ = 0.06	*Cramer’s V* = 0.06
From 1.200 to 1.800 €	7.00 (1)	20.0 (3.00)		
From 600 to 1.200 €	33.0 (5)	67.0 (10.0)		
Less of 600 €	53.0 (8)	13.0 (2.00)		
**Pain duration (years)**				
Mean (SD)	26.1 (14.8)	-	-	-
Range	8.00–50.0	-	-	
**Diagnosis time (years)**				
Mean (SD)	10.7 (5.84)	-	-	-
Range	5.00–27.0	-		
**Time elapsed since the diagnosis (years)**				
Mean (SD)	15.5 (13.1)	-	-	-
Range	0.00–40.0	-		
Pain intensity (10 cm VAS) *				
Mean (SD)	4.35 (2.14)	0.41 (1.10)	*t* = 6.35	*d* = 2.32
Range	0.70–8.00	0.00–4.00		
Fatigue level (10 cm VAS) *				
Mean (SD)	5.15 (2.38)	1.70 (1.60)	*t* = 4.65	*d* = 1.70
Range	1.00–9.10	0.00–3.90		
Sleep quality level (10 cm VAS) *				
Mean (SD)	5.99 (2.40)	2.46 (2.61)	*t* = 3.85	*d* = 1.41
Range	0.90–10.0	0.00–7.10		
**Medications % (n)**				
Analgesics *	53.0 (8)	0.00 (0.00)	χ^2^ = 0.001	*Cramer’s V* = 0.001
NSAIDs	13.0 (2)	0.00 (0.00)	χ^2^ = 0.14	*Cramer’s V* = 0.14
Anxiolytic *	47.0 (7)	0.00 (0.00)	χ^2^ = 0.003	*Cramer’s V* = 0.003
Antidepressants *	67.0 (10)	7.00 (1.00)	χ^2^ = 0.001	*Cramer’s V* = 0.001
Antiepileptics	7.00 (1)	0.00 (0.00)	χ^2^ = 0.31	*Cramer’s V* = 0.31
Antipsychotics	0.00 (0)	7.00 (1.00)	χ^2^ = 0.31	*Cramer’s V* = 0.31

*Note. SD* = standard deviation; *VAS* = visual analogue scale; *NSAIDs* = non-steroidal anti-inflammatory drugs; * *p* <.05

### Instruments

#### Semi-structured interview

A semi-structured interview was conducted to collect individual and clinical data and to confirm inclusion/exclusion criteria. The visual analogue scale was used to evaluate pain intensity, sleep quality and fatigue. This interview was used in another previously published study [28]. The interview guide is in the supplementary material.

#### Beck depression inventory (BDI) [42]

The BDI is a self-report inventory to assess current depressive symptoms. It is composed by 21 items, and the answer is given on a 4-point Likert scale (0 = non-depressing state; 3 = severe depression). This instrument in Portuguese presents good psychometric qualities, for main sample α = 0.91; for student sample α = 0.895; for clinical sample α = 0.925.

#### Fibromyalgia impact questionnaire (FIQ;) [43]

The FIQ provides measures of the health-related status and functional capacity of patients with fibromyalgia. It is composed by 20 questions that explore the patient’s functional ability to perform daily tasks (cooking, cleaning, walking, mobility, among others). Responses are distributed on a Likert scale of 0 (able to always) to 3 (unable to do). The answer is given on a 4-point Likert scale (0 = always perform; 3 = unable to perform). The FIQ in Portuguese presents good psychometric qualities (α = 0.814).

#### Pain catastrophizing scale (PCS) [44]

The PCS is a self-report questionnaire regarding thoughts, perceptions, and feelings related to pain. It is composed by 13 items and participants are instructed to indicate the frequency of the described symptoms in a 5-point Likert scale (0 = never; 4 = always). The PCS in Portuguese presents good psychometric qualities, for rumination scale α = 0.796; for magnification scale α = 0.789; for discouragement scale α = 0.897.

### Tasks

#### Oddball dual task

**Fig. 1:**
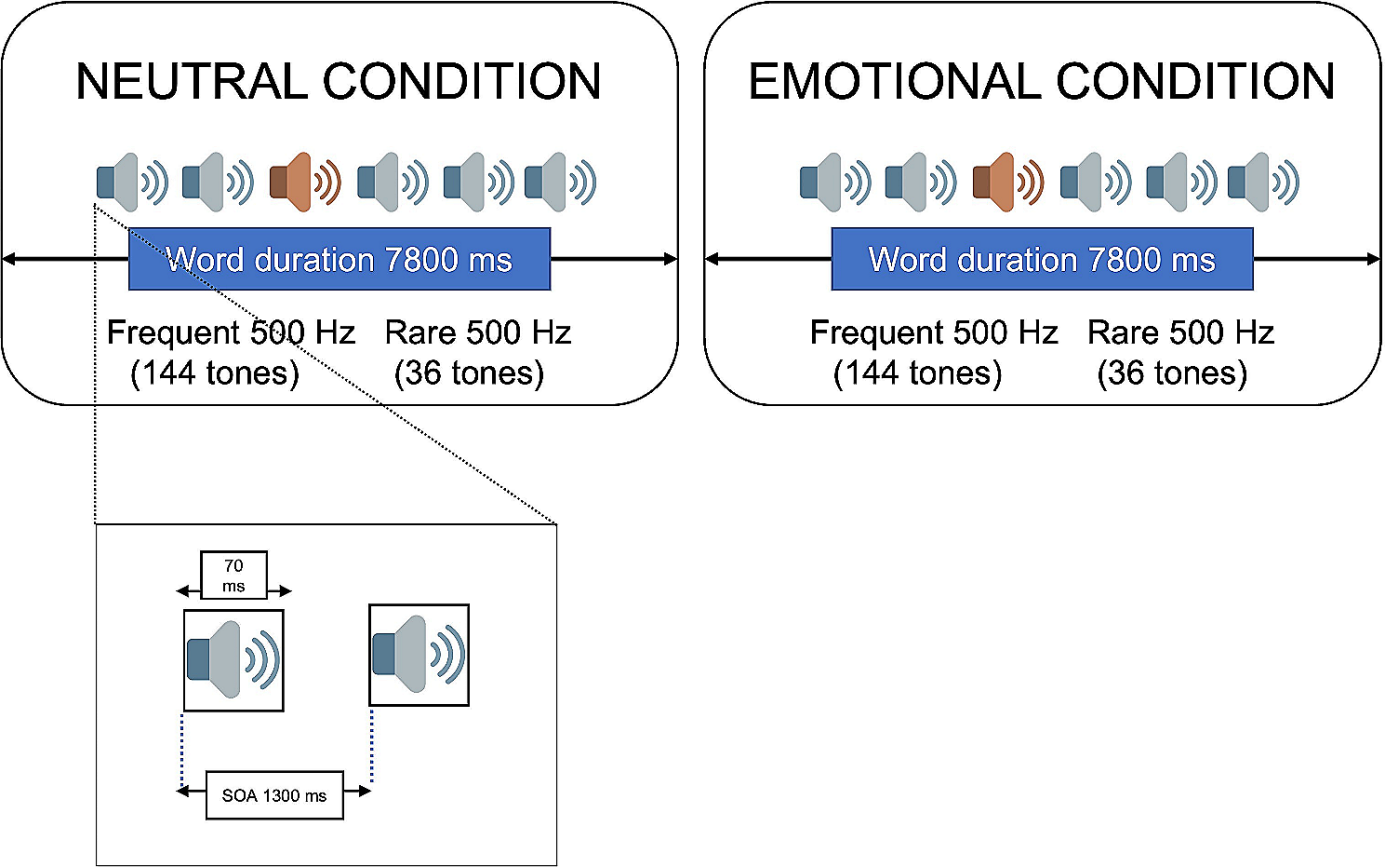
Schematic representation of oddball dual task

In the present study, the oddball paradigm consisted of a dual-task version. The oddball task was composed of two tones, a 500 Hz tone with 80% probability of occurrence (frequent trials), and a 1500 Hz tone with 20% probability of occurrence (rare trials). The duration of each stimulus was 70 ms and the interval between the onset of the tone and the next one was 1300 ms. The task was composed of two blocks of 180 trials each. Rare tones were not presented twice in a row. The tones were presented through two headphones on either side of the participant. The second task was composed of word stimuli presented simultaneously to the oddball task. These words could be neutral (irrelevant condition) or emotional (related to pain; relevant condition), and its occurrence was synchronized with the presentation of the tones. The visual stimuli were composed of 30 neutral words and 30 pain-related words repeated twice. The words were written in white, on a black background, and were presented on a 17-inch monitor during 7800 ms (Fig. 1). The task was built on E-prime 2.0 (2011, Psychology Software Tools, Inc., Sharpsburg, PA, USA). Before the two experimental blocks, a training block of four trials was performed to ensure that participants understood the task. Participants were instructed to identify the rare stimuli by pressing a button on a keyboard, while reading the words mentally. After the task, participants were asked to recognize the neutral and the pain-related words form a list. The hits and the reaction time of the responses were recorded. See the list of verbal stimuli in Table S1.

#### Emotional stroop task

**Fig. 2:**
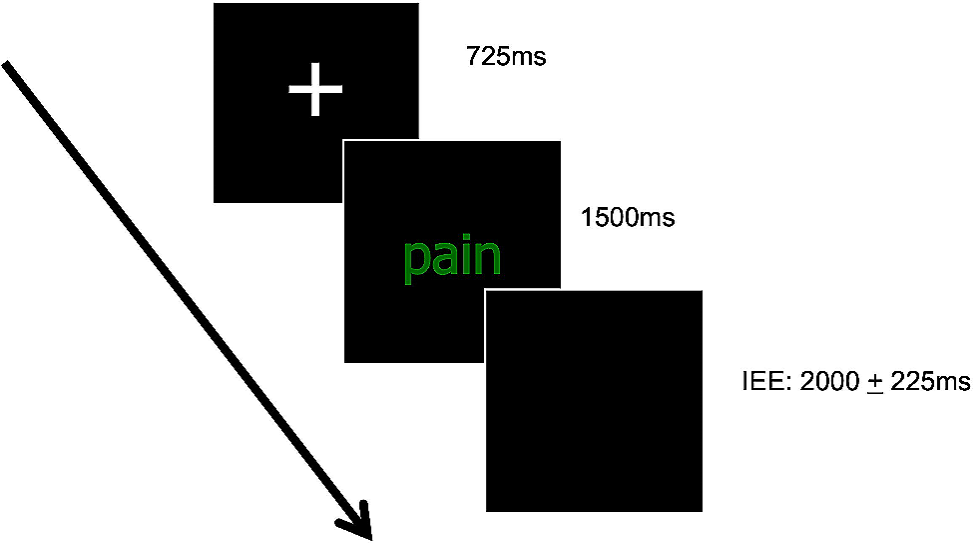
Schematic representation of Emotional Stroop Task

The Emotional Stroop Task was also selected to investigate the effect of pain-related words as distractors. The rational is that the content of the words should require the attentional resources of the participants, delaying the task goal of colour identification [30]. The task consisted of the presentation of relevant (pain-related) and neutral words in different colours (blue, green, red, and yellow) on a black background. The task was built on E-PRIME 2.0 software (2011, Psychology Software Tools, Inc., Sharpsburg, PA, USA) and it was composed of 64 trials divided in eight experimental blocks of eight sequenced trials each. The task started with a training block of four trials, followed by 4 blocks composed of pain-related words and four blocks composed of neutral words. Each trial started with a fixation white cross (725 ms), followed by a word (1500 ms) and an inter-trial interval (IEE) that varied randomly between 1775 and 2225 ms (Fig. 2). In each block, each sequenced colour was randomly submitted twice, since the same colours could not be repeated in sequence. The participants were instructed to identify the colour of the words as quickly as possible by pressing one of the buttons on a keyboard containing the four response alternatives. The hits and the reaction time of the responses were recorded.

### Procedures

The current study was part of a larger research project [45] and was conducted in accordance with the Helsinki Declaration and was approved by the local Research Ethics Committee. Participants were tested individually in one experimental session conducted in a laboratorial setting. After the informed consent, the semi-structured interview was conducted. The BDI, FIQ, and PCS were then administered in a balanced order. Participants who fulfilled the inclusion criteria were recruited for experimental tasks, which was performed inside an EEG chamber, which were administered in a balanced order after the placement of the EEG cap.

### EEG recording and processing

The electroencephalographic (EEG) data were recorded using a 128-electrode Hydrocel Geodesic Sensor Net, a NetAmps 300 amplifier (both from Electrical Geodesics Inc., Eugene, EUA) and a digitizing rate of 500 Hz. Impedances were kept below 50 kOhm for all electrodes, as this is a high impedance system. The electrodes were referenced to Cz during recording and re-referenced offline to the average of electrodes placed on the left and right mastoids. The EEG data was pre-processed in EEGLAB (version 2021.0) as a toolbox of MATLAB 2017b. The data were downsampled to 250 Hz and band-pass filtered at 0.1–30 Hz. Bad channels were interpolated (up to a maximum of 10% of the sensors), and data were decomposed through Independent Components Analysis. Eye-blink, saccade and heart rate artifacts were corrected by subtracting the respective component activity from the signal. The EEG records were segmented into epochs ranging from − 200 to 800ms, time-locked to the tone in the Oddball Dual Task and to the word in the Emotional Stroop Task. All segments were visually inspected after baseline correction (200ms pre-stimulus), and the remaining artefactual epochs were manually rejected. The mean percentage of artefact rejection procedure for each condition was 10%. Epochs were averaged by condition (Oddball dual-task: emotional– rare, neutral– rare, emotional– frequent, and neutral frequent; emotional Stroop task: emotional and neutral).

In the Oddball Dual Task, two ERP components were analysed for each participant, the N100 and the P300. The time window of each ERP component was defined based on the grand average of each group, considering that patients with FM may have an increased peak latency [25]. Thereby, according to the grand average of each group, the N100 was quantified as the peak amplitude in the time window of 70–170 ms for both groups, but the P300 was quantified as the mean amplitude in the time window of 280–380 ms for the HC group and 320–420 for the FM group. Based on previous literature, as well as the inspection of topographical maps, one region where peaks were most prominent (maximum negative/positive voltage) were selected for peak scoring. Thereby, the N100 and P300 were measured at Pz cluster (electrodes 54 55 61 62 78 79) [34].

In the emotional Stroop task, we analysed the P200 for each participant. According to the grand average and topographical maps of each group, the P200 was quantified as the peak amplitude in the time window of 130–230 ms for the HC group and 150–250 for the FM group, at Fz cluster (electrodes 4, 5, 10, 11, 12, 16, 18, 19).

### Statistical analysis

The results obtained during the Oddball Dual Task (hits, false alarms, omissions) were analysed through repeated-measures ANOVAs, with group (FM, HC) as between-participants factor, and condition (emotional, neutral) as within-participant factors. For the electrophysiological results, amplitudes and latencies were analysed through repeated-measures ANOVAs, with group (FM, HC) as between-participants factor, and frequency (frequent, rare) and condition (emotional, neutral) as within-participant factors.

For the electrophysiological results obtained during the Emotional Stroop Task, amplitudes and latencies of the P200 were analysed through repeated-measures ANOVAs, with *group* (FM, HC) as between-participants factor, and *condition* (emotional, neutral) as within-participant factors. This model was used to analyse reaction times and accuracy rates.

ANCOVAs were also performed to explore the effect of depression, anxiety, and pain catastrophizing on behavioral results of the tasks. Pearson’s *r* was computed to explore the correlations between behavioral and electrophysiological results. The threshold for statistical significance was set at α = 0.05 for all analyses. Violations of sphericity were corrected via the Greenhouse-Geisser method. Significant ANOVA main effects were quantified using Bonferroni-corrected post-hoc comparisons. Statistical analysis was performed using SPSS 24 (IBM Corp., Armonk, NY, USA).

## Results

### Behavioral results

Significant differences were observed between groups in depression (BDI), *t*(28) = 5.50, *p* <.001, *d* = 2.01, and pain catastrophizing (PCS), *t*(28) = 26.2, *p* <.001, *d* = 1.26. Results showed that FM group had higher values in all of the self-report measures, as shown in Table 2. Covariance analyses were performed to explore the effect of the above variables on the results of both experimental tasks, and non-significant differences were found (all *p* >.05).

**Table 2:** Self-reported measures of depression, fibromyalgia impact and pain catastrophizing for fibromyalgia and healthy control groups

	Fibromyalgia Mean (SD) (*n* = 15)	Healthy controls Mean (SD) (*n* = 15)
Depression (BDI, total) *	20.9 (9.87)	5.20 (5.05)
Pain catastrophizing (PCS, total) *	31.8 (16.5)	13.4 (12.5)

*Note*. * *p* <.001, SD = Standard deviation

Regarding the hits obtained during the Oddball Dual Task (Table 2), we did not find main effect of *group F*(1,28) = 3.215, *p* =.084, η^2^_p_ = 0.103, *condition F*(1,28) = 0.321, *p* =.576, η^2^_p_ = 0.011, nor a significant group*condition interaction (*F* < 1). The analyses performed for false alarms did not reveal significant main effects of *group F*(1,28) = 2.362, *p* =.136, η^2^_p_ = 0.078, *condition* (F < 1), nor significant group*condition. The analysis of omissions revealed the same pattern of results: we did not find main effects of group, *F*(1,28) = 3,128, *p* =.088, η^2^_p_ = 0.101, condition (*F* < 1), nor a significant group*condition interaction, (*F* < 1). Regarding reaction times to the hits (see Table 2), no main effects were found for *Group* or *Condition*, nor for the interaction between both variables (all *F* < 1).

Regarding the hits obtained during the Emotional Stroop Task, we found a main effect of *group*, *F*(1,27) = 8.067, *p* =.008 η^2^_p_ = 0.230, revealing that healthy controls had higher hits than patients with FM (Table 2). The main effect of *condition* was non-significant *F*(1,27) = 2.529, *p* =.123 η^2^_p_ = 0.086, along with the group*condition interaction (*F* < 1). Regarding reaction times (see Table 3), we found a main effect of group, *F*(1,27) = 13.707, *p* =.001 η^2^_p_ = 0.337, showing that patients with FM had higher reaction times than healthy controls. The main effect of *condition* was non-significant *F*(1,27) = 1.291, *p* =.266 η^2^_p_ = 0.046, along with the group*condition interaction, *F*(1,27) = 1.116, *p* =.300 η^2^_p_ = 0.040.

**Table 3:** Descriptive statistics of the results obtained in Oddball Dual Task and Emotional Stroop Task

	Fibromyalgia Mean (SD) (*n* = 15)	Healthy controls Mean (SD) (*n* = 15)
**Oddball Dual Task**		
Hits - Emotional (%)	93.65 (16.19)	99.26 (1.27)
Hits - Neutral (%)	95.18 (5.60)	99.44 (1.15)
False Alarms - Emotional (%)	3.38 (11.18)	0.42 (0.51)
False Alarms - Neutral (%)	2.59 (2.45)	0.42 (0.51)
Omissions - Emotional (%)	6.54 (16.89)	0.74 (1.27)
Omissions - Neutral (%)	4.82 (5.60)	0.56 (1.15)
Reaction Time - Emotional (ms)	445.10 (151.63)	405.81 (103.77)
Reaction Time - Neutral (ms)	419.49 (114.78)	413.85 (87.27)
**Emotional Stroop Task**		
Accucary - Emotional (%)	87.95 (11.75)	95.00 (3.88)
Accucary - Neutral (%)	89.96 (7.37)	96.67 (2.76)
Reaction Time - Emotional (ms)	953.57 (152.34)	767.66 (140.02)
Reaction Time - Neutral (ms)	952.76 (170.63)	745.38 (116.17)

*Note*. SD = Standard deviation

## Electrophysiological results

### Oddball dual task

#### N100

**Fig. 3:**
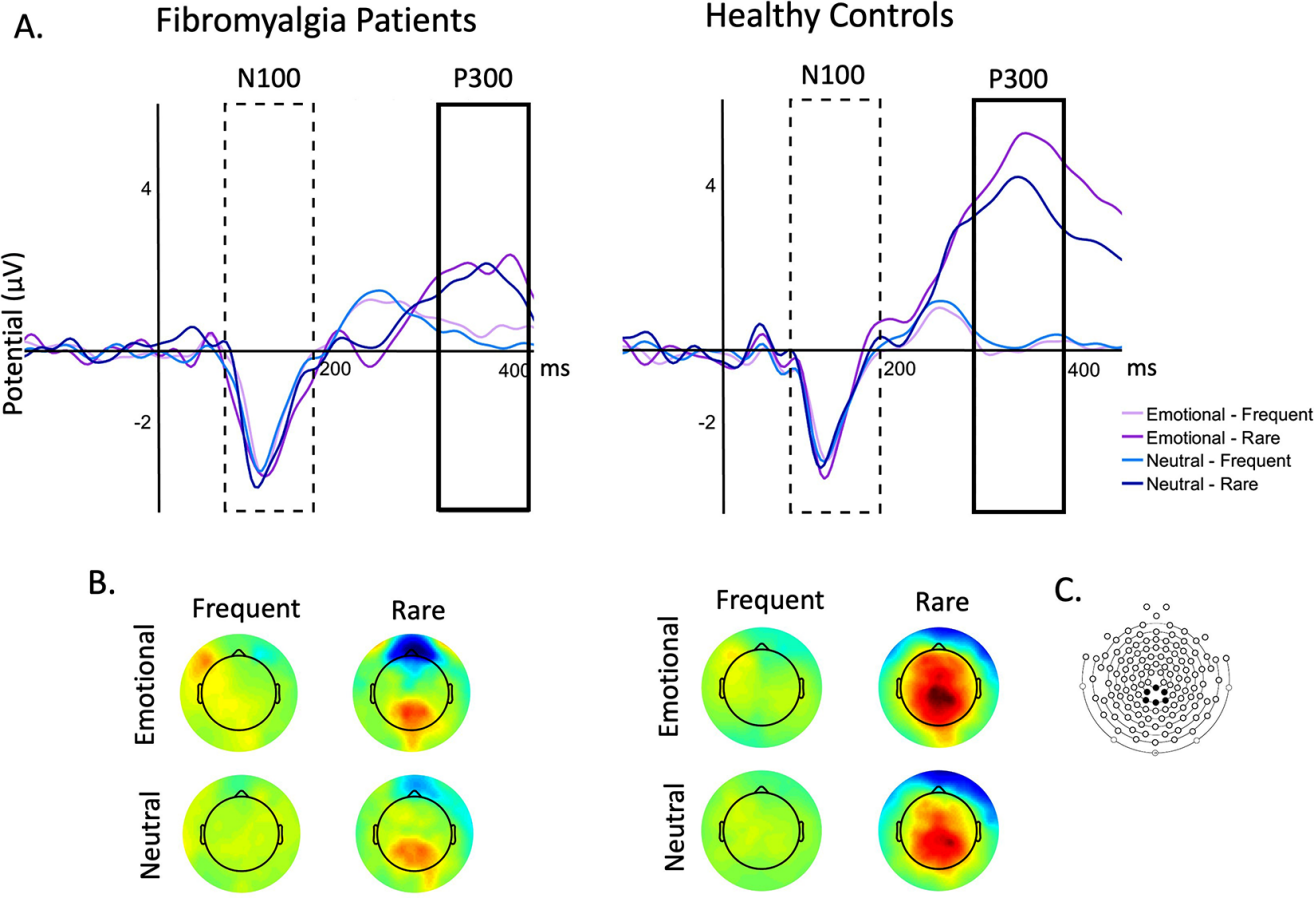
N100 and P300 obtained in Oddball Dual Task. Note. (**A**) Grand-average of N100 and P300 for patients with fibromyalgia and healthly controls. The N100 was quantified as the peak amplitude in the time window of 70–170 ms for both groups, while the P300 was quantified as the mean amplitude in the time window of 280–380 ms for the healthy controls and of 320–420 for patients with fibromyalgia. (**B**) Topographical maps for event-related potentials elicited by all conditions (neutral, emotional, frequent, and rare). (**C**) Electrode locations in the 128-channel HydroCel Geodesic Sensor Net (EGI) where event-related-potential components were measured

We found a main effect of *frequency*, *F*(1, 27) = 13.10, *p* =.001, η^2^_p_ = 0.327, revealing that rare stimuli elicited higher peak amplitudes than frequent stimuli. The main effect of *group* was not significant, *F*(1, 27) = 1.61, *p* =.216, η^2^_p_ = 0.056, along with the main effect of *condition*, *F*(1, 27) = 0.458, *p* =.504, η^2^_p_ = 0.017. We did not find significant interactions (all *p*s > 0.181). Regarding the N100 latency, we did to find a main effect of *group*, *F*(1, 27) = 1.61, *p* =.216, η^2^_p_ = 0.056, but the main effect of *condition* was marginally significant, *F*(1, 27) = 3.68, *p* =.066, η^2^_p_ = 0.120. The main effect of *frequency* was non-significant, *F*(1, 27) = 0.13, *p* =.910, η^2^_p_ = 0.000, along with all the interactions (all *p*s > 0.181) (Fig. 3).

#### P300

We found a main effect of *group*, *F*(1, 27) = 5.11, *p* =.032, η^2^_p_ = 0.159, revealing that patients with FM had lower amplitudes than healthy participants. We also found a main effect of *frequency*, *F*(1, 27) = 42.22, *p* <.001, η^2^_p_ = 0.610, revealing that rare stimuli elicited higher mean amplitudes than frequent stimuli. Moreover, we found a significant group*frequency interaction, *F*(1, 27) = 8.25, *p* =.008, η^2^_p_ = 0.234, revealing that rare stimuli elicited higher mean amplitudes than frequent stimuli for healthy controls (*p* =.010), but this comparison was non-significant for patients with FM (*p* =.473). The main effect of *emotion* was non-significant, *F*(1, 27) = 1.41, *p* =.245, η^2^_p_ = 0.050, along with the remaining interactions (all *p*s > 0.219) (Fig. 3).

### Emotional stroop task

#### P200

**Fig. 4:**
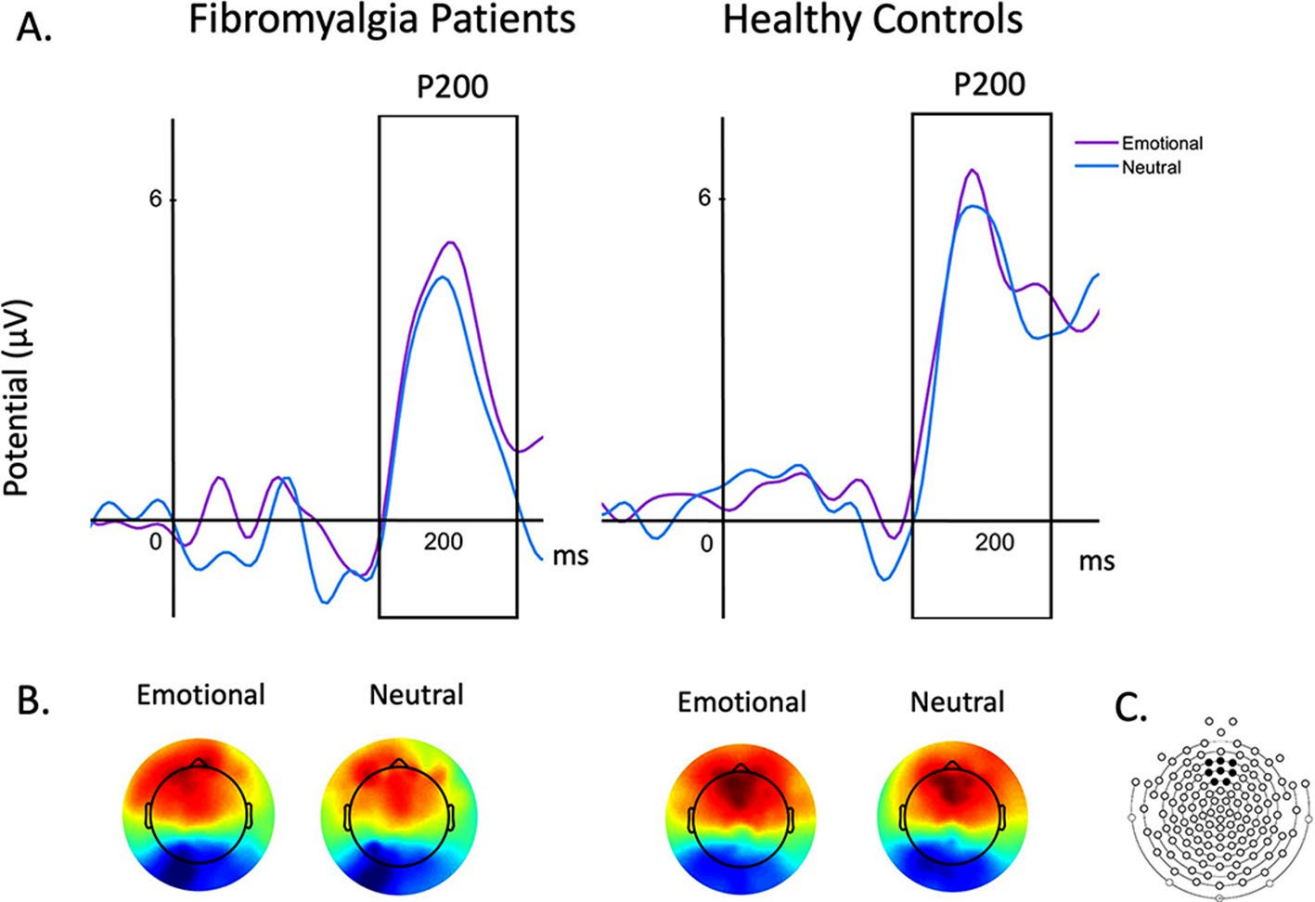
P200 obtained in emotional stroop task. Note. (**A**). Grand-average of P200 for patients (150–250 ms) with fibromyalgia and healthly controls (130–230 ms). (**B**) Topographical maps for event-related potentials elicited by emotional and neutral conditions. (**C**) Electrode locations in the 128-channel HydroCel Geodesic Sensor Net (EGI) where event-related-potential components were measured

Regarding the P200 peak amplitude (Fig. 4), we did not find a main effect of *group*, *F*(1, 26) = 0.641, *p* =.431, η^2^_p_ = 0.024, *condition, F*(1, 26) = 0.252, *p* =.620, η^2^_p_ = 0.010, nor a significant group*condition interaction, *F*(1, 26) = 0.121, *p* =.731, η^2^_p_ = 0.005. Regarding the P200 latency, we found a main effect of group, *F*(1, 26) = 4.378, *p* =.046, η^2^_p_ = 0.114, showing that patients with FM had higher latencies than healthy controls. The main effect of *condition*, *F*(1, 26) = 2.87, *p* =.102, η^2^_p_ = 0.099, and the interaction group*condition were non-significant, *F*(1, 26) = 0.616, *p* =.440, η^2^_p_ = 0.023.

Descriptive statistics of ERP amplitudes and latencies for both tasks are available in Table 4.

**Table 4:** Means (standard deviations) for N100, P300 and P200

Oddball Dual Task		Fibromyalgia		Healthy controls	
		Amplitude	Latency	Amplitude	Latency
		*n* = 15		*n* = 14	
N100	Emotional - Frequent	-3.61 (1.78)	125.07 (15.07)	-2.96 (1.52)	123.24 (8.06)
	Emotional - Rare	-4.57 (1.91)	124.36 (13.72)	-3.54 (2.59)	119.10 (7.69)
	Emotional - Frequent	-3.56 (1.70)	118.67 (11.91)	-2.99 (1.54)	118.38 (7.93)
	Emotional - Rare	-4.40 (2.12)	123.82 (14.00)	-3.23 (2.32)	117.33 (9.40)
P300	Emotional - Frequent	0.61 (0.94)	-	0.09 (0.84)	-
	Emotional-Rare	2.19 (2.79)	-	4.74 (2.17)	-
	Emotional - Frequent	0.28 (1.47)	-	0.30 (0.81)	-
	Emotional-Rare	1.85 (3.03)	-	3.81 (1.97)	-
Emotional Stroop Task		*n* = 14		*n* = 14	
P200	Emotional	7.22 (3.78)	198.39 (14.83)	8.15 (4.23)	188.71 (14.81)
	Neutral	6.76 (2.39)	204.43 (17.11)	8.06 (4.97)	190.93 (17.11)

*Note*. Amplitudes are presented in *µV* and latencies in milliseconds

## Discussion

Chronic pain may be associated to a negative attentional bias in the processing of pain-related information, which may initiate, exacerbate, and maintain the characteristics of a given disease, as well as de processing of a painful stimuli. However, the results regarding this hypothesis are inconsistent. Studies have shown that patients with chronic pain selectively process information related to their clinical condition [24, 46], but other studies have found no attentional bias [47, 48]. Probably, the inconsistent results found so far can be explained differences in the methodologies of each study, such as the type of stimuli.

The present study aimed to expand this knowledge, investigating this hypothesis through an Oddball Dual Task and an Emotional Stroop Task adapted to an ERP methodology. Specifically, we investigated the existence of an attentional bias for neutral and pain-related verbal stimuli in patients with FM, compared to healthy female controls. With this methodology, we tested the following hypothesis: fibromyalgia is characterized by an attentional bias toward negative, pain-related information, resulting in larger ERP components for the pain-related condition compared to the neutral condition. For the control group, we expect to find similar amplitudes for both conditions.

The results of both tasks were consistent but did not support this hypothesis. Specifically, in the Oddball Dual Task, we found significant differences in the amplitude of P300 between both groups, showing that female patients have lower amplitudes than controls. We did not find a group by condition interaction, but we found that patients with FM had similar amplitudes for rare and frequent stimuli instead of the typical oddball effect [49] that was found for controls. Thereby, this result is suggestive of a nonspecific deficit in sustained attention in FM, rather than attentional bias towards pain-related stimuli compared to the neutral ones. This reduced sustained attention may be translated into an inability to distinguish relevant from irrelevant information according to the goals of the task. This may suggest that both conditions demand greater attentional resources from people with FM, thus indicating general difficulties in attentional processing. This result is consistent with the findings of two previous studies conducted with patients with FM, which found a reduced amplitude of P300 that was interpreted as an attentional deficit [25, 26].

The results of the Emotional Stroop Task support this interpretation as we found that patients had higher reactions times and less hits than controls. For this task, we also found an increased P200 latency for patients than controls. Taken together, these results may suggest that an attentional deficit and decreasing processing speed, being less efficient in processing the relevant task (naming colors) and ignoring the content of the words (irrelevant stimuli).

On note, the neural and behavioral results found in our tasks are in line with several neuropsychological studies that revealed significant deficits in sustained attention and processing speed in patients with FM [50, 51]. Moreover, the lack of emotional modulation was previously found in previous studies with patients with FM that used implicit emotional processing tasks. For instance, a lack of emotional interference in patients was also seen in the emotional variant of the Stroop test [14, 52] and the picture frame task [53].

However, a study conducted using a dot-probe task [29] found lower latencies for P2 for pain-related faces, i.e., contrary to our findings. These different results may be because the stimuli used were visual, which can elicit faster responses (the processing speed is faster) than reading the words, and because they involve different information processing neural circuits. Also, to the fact that the task is different, since the Stroop task– which we have used– has been related to the measurement of cognitive interference and the dot-probe task has been related to a pure measure of selective attention [54].

Another study [9] found larger frontal ERP amplitudes (P450) in an emotional Stroop task. These different results from ours, despite using the same task, may be because we have used several emotional conditions (fibromyalgia symptoms, negative arousing words, positive arousing, and neutral words), whereas our study measured two conditions; words related to pain and neutral words. It is worth mentioning that the verbal stimuli vary in terms of language, the first was in Spanish, ours was in Portuguese. That is the methodological difference in that the task can provoke different responses at a neuropsychophysiological level. However, the authors relate the greater amplitudes of frontal ERPs (P450) to dysfunctional attentional mechanisms causing enhanced and dysfunctional effort of processing. This interpretation coincides with our evidence that there may be an attentional deficit in FM.

Attentional bias training of fibromyalgia patients has also been tested [29]. The study demonstrated that after training, there was an overall reduction in the amplitude of the P2 component followed by an improvement in the N2a amplitude for the ABM condition compared to the control condition. Studies such as these support our findings, but more studies related to the neural mechanisms underlying cognitive processing in FM are required.

We also investigated the effects of affective variables, such as depression, impact of FM, and pain catastrophizing on participants’ performance in both tasks. As expected, people suffering from FM a reported higher depression symptomatology, as well as higher scores on pain-related scales. While certain studies based on neuropsychological tests of attention demonstrate that anxiety and depression do not contribute to attentional deficits [55], other studies using, for instance the Stroop task showed that anxiety and depression modulates attentional bias [9, 24, 55].

In the present study, although patients have higher levels of depression than controls, as well as thoughts, perceptions, and feelings related to pain, these variables did not influence the results of both tasks. This is consistent with previous findings that did not find comorbid symptomatology to affect the cognitive performance of the same or different tasks in our study [9, 24, 46, 55–57]. The results of these variables did not appear to influence the results of both tasks. According to these results, we can infer FM is associated with higher levels of depression, as well as thoughts, perceptions, and feelings related to pain, but no influence on cognitive performance in these patients.

Despite the novelty of the results, several limitations must be considered during the interpretation of our findings. The sample size is small, which may limit the statistical power as well as the generalization of the results. Not having a clinical control group with another type of chronic pain prevents us from concluding that the attention deficits suggested by our results are characteristic of FM. The exposure time of the stimuli may have been too short to produce the effects of interest. The use of verbal stimuli can cause motor artifacts, and words are an abstraction of pain. They may not evoke a similar response as somatosensory pain-related stimuli and are susceptible to producing motor artefacts. For future studies, it is recommended to include a block of pain-related non-verbal and verbal stimuli, and collect physiological data to increase understanding of behavioral outcomes. For future studies, it would be interesting to establish multiple regression models to study the associations between clinical variables - including pain severity - and task performance in the FM group. Despite these limitations, this study is a further step in the direction of a better understanding of the cognitive alterations associated with FM, opening new directions for future research in this area.

## Conclusion

As far as we know, this is the first study providing data on attentional functioning in people with chronic pain with two tasks, Oddball Dual-Task and Emotional Stroop Task. It seems feasible that patients with chronic pain do not present attentional biases, but a general alteration of attentional functioning. People with FM require greater cognitive effort to perform the tasks, which coincides with what was reported by the patients. ERPs data seem to show a general alteration of information processing, but in simple tasks it seems to be compensated with automatic attentional resources.

### Electronic supplementary material

Below is the link to the electronic supplementary material.

Supplementary Material 1

Supplementary Material 2

## Data Availability

Data cannot be provided because their availability was not written consented by the participants. However, the data can be provided to the reviewers if requested. Data and materials are available on request from the corresponding author.
