# Supplementary material for: Attentional deficits in fibromyalgia: an ERP study with the oddball dual task and emotional stroop task
Source: BMC Psychol. 2024 Feb 29;12:104. doi: 10.1186/s40359-024-01601-3 (PMC10902965; doi:10.1186/s40359-024-01601-3)
Supplement: Supplementary file 1 — Supplementary Material 1 [file 40359_2024_1601_MOESM1_ESM.docx]

**INTERVIEW GUIDE**

**DEMOGRAPHIC DATA: Date:** _______________________

**Surname and First Name** : _____________________________________________________

**Age (years):** _____________ **Place of birth (city, country):** ______________________

| **Education:** |  | **Civil status:** |  |
| --- | --- | --- | --- |
| Primary  Basic cycle  High school  Higher education | (1)  (2)  (3)  (4) | Married  Single  Widower  Separated/Divorced | (1)  (2)  (3)  (4) |
| **Employment status:**  Active  Never Active  Inactive more than 1 year  Inactive less than 1 year | (1)  (2)  (3)  (4) | **Salary (monthly)**  More than €1,800  From €1,200 to €1,800  From €600 to €1,200  Less than €600 | (1)  (2)  (3)  (4) |

**Are you right or left handed?** ________________________________________________

| **Mother Language:** □ Spanish □ Other Which one? | **Do you speak other languages?** □ No □ Yes Which one? |
| --- | --- |
| **Are you bilingual?** □ No □ Yes  Which? |  |

**When did you first have symptoms of pain?** Please indicate the approximate year and month:

**When were you diagnosed with Fibromyalgia?** Please indicate the approximate year and month:

**Briefly describe your pain** , including especially adjectives that are characteristic of your pain experience:

**Current medication use:**

|  |
| --- |

Next, draw a mark (vertical line) at the location on the line that best reflects your **DEGREE OF PAIN AND FATIGUE AT THIS VERY MOMENT:**

*No pain*

*Worst possible pain*

**PAIN**

*No fatigue*

*Worst possible fatigue*

**FATIGUE**

**How many hours did you sleep last night?** Enter the total number of hours: __________

Next, draw a mark (vertical line) at the place on the line that best reflects the **QUALITY OF YOUR SLEEP ON THE LAST NIGHT** :

**SLEEP**

*The worst possible quality of sleep*

*The best quality of sleep*
