# Supplementary material for: Attentional deficits in fibromyalgia: an ERP study with the oddball dual task and emotional stroop task
Source: BMC Psychol. 2024 Feb 29;12:104. doi: 10.1186/s40359-024-01601-3 (PMC10902965; doi:10.1186/s40359-024-01601-3)
Supplement: Supplementary file 2 — Supplementary Material 2 [file 40359_2024_1601_MOESM2_ESM.docx]

Table S1. *Relevant (pain-related) and neutral words in Oddball and Stroop task*

| **Portuguese**  **Pain-related words** | **English**  **Pain-related words** | **Portuguese**  **Neutral words** | **English**  **Neutral words** |
| --- | --- | --- | --- |
| apavaronte | appalling | aciganada | stunned |
| tensa | tense | acromática | achromatic |
| apertada | tight | adiposa | adipose |
| maçadora | dull | adoçante | sweetening |
| enlouquecedora | maddening | aquática | aquatic |
| castigante | punishing | aquosa | watery |
| forte | strong | azul | blue |
| escaldante | scorching | balsámica | balsamic |
| penetrante | penetrating | barbuda | bearded |
| miserável | miserable | básica | basic |
| mortificante | mortifying | binocular | binocular |
| exaustiva | exhaustive | bissemanal | bi-weekly |
| cortante | sharp | bissexta | leap year |
| cruel | cruel | bovina | bovine |
| sensível | sensitive | cardinal | cardinal |
| desgastante | exhausting | cartesiana | Cartesian |
| torturante | torturous | celta | Celtic |
| aguda | sharp | civil | civil |
| magoada | hurt | conceptiva | conceptive |
| agonizante | agonizing | convocatória | call |
| insuportável | unbearable | cosmopolítica | cosmopolitical |
| intensa | intense | decorativa | decorative |
| atormentante | tormenting | descapotável | convertible |
| cansativa | tiring | digital | digital |
| incómoda | uncomfortable | etária | age |
| amedrontadora | frightening | ética | ethical |
| pavorosa | dreadful | extrínseca | extrinsic |
| sufocante | suffocating | sinfónica | symphonic |
| dolorida | painful | triangular | triangular |
| maldita | cursed | umbilical | umbilical |
